# Legal Literacy in Clinical Nursing Practice: A Walker and Avant Concept Analysis

**DOI:** 10.3390/nursrep16060200

**Published:** 2026-06-12

**Authors:** Yufei Xing, Xiaolong Wang, Enming Zhang, Jiajia Yu, Qiong Fang

**Affiliations:** 1 School of Nursing, Shanghai Jiao Tong University, Shanghai 200025, China; xingyufei@sjtu.edu.cn (Y.X.); w.xg.00@sjtu.edu.cn (X.W.); zhangenming@sjtu.edu.cn (E.Z.); 2 KoGuan School of Law, Shanghai Jiao Tong University, Shanghai 200240, China; jiajiay@sjtu.edu.cn

**Keywords:** clinical nurses, legal literacy, concept analysis, nursing management, Walker and Avant

## Abstract

**Background:** The legal dimensions of nursing practice have become increasingly complex, yet the concept of legal literacy in clinical nurses remains insufficiently defined. Existing studies use terms such as legal knowledge, legal awareness, legal cognition, and law-based practice capacity inconsistently, which hinders conceptual clarity, valid measurement, and targeted educational intervention. This study aimed to clarify the conceptual boundaries, defining attributes, antecedents, consequences, empirical referents, and operational definition of legal literacy in clinical nurses. **Methods:** A concept analysis was conducted using Walker and Avant’s eight-step method. A systematic literature search was performed across six databases and supplemented by searches in JSTOR and HeinOnline for non-clinical uses of the concept. The search covered database inception to December 2024. Screening and reporting followed PRISMA 2020 guidelines. Fifty-six papers were included. Data extraction and analysis were conducted using content analysis with independent dual-reviewer coding. **Results:** Legal literacy in clinical nurses was distinguished from four related concepts: legal knowledge, legal awareness, legal cognition, and medical ethics. Three defining attributes were identified: normative understanding, value internalization oriented toward rights and responsibilities, and law-based situational practice. Antecedents were identified at macro, meso, and micro levels, while consequences were observed for individual nurses, healthcare organizations, and patient rights. Analysis of empirical referents revealed a persistent gap between conceptualization and measurement, particularly in assessing law-based situational practice. An operational definition was developed accordingly. **Conclusions:** Legal literacy in clinical nurses is a multidimensional professional competency integrating legal understanding, rights- and responsibility-oriented value internalization, and the ability to translate these into lawful clinical action. The findings provide a conceptual basis for future instrument development and targeted educational and management interventions.

## 1. Introduction

The legal dimensions of nursing practice have become increasingly complex as nurses’ roles have expanded beyond task-based care to include autonomous clinical decision-making, care coordination, advanced practice, and patient advocacy [1,2]. In contemporary healthcare settings, nurses routinely work at the intersection of clinical care and legal accountability. Their responsibilities include executing medical orders, facilitating informed consent, maintaining legally defensible documentation, managing adverse events, protecting patient privacy, and communicating with patients and families in situations with potential medicolegal consequences [2,3,4]. Accordingly, understanding and applying legal norms is no longer a peripheral professional requirement, but a constitutive element of competent nursing practice.

Evidence from multiple jurisdictions suggests that deficiencies in nurses’ legal knowledge and law-based practice carry direct consequences for patient safety and professional accountability. A cross-sectional survey of health professionals in Australia found that a substantial proportion lacked accurate knowledge of the law governing end-of-life decisions, with nurses demonstrating particularly significant gaps in legally regulated areas such as withholding treatment, substitute decision-making, and the doctrine of double effect [5]. A separate qualitative study confirmed that nurses’ legal uncertainty in palliative care settings contributed to the undertreatment of pain and symptom distress, as fear of legal repercussions deterred appropriate clinical action [6]. In primary care contexts in China, clinicians’ legal literacy has been positively associated with the quality of healthcare delivery, with lower levels linked to increased risk events and weaker procedural compliance [7]. At the level of professional liability, recent insurance data indicate that the average incurred cost per nursing malpractice claim continues to rise, with communication failures, documentation deficiencies, and deviations from procedural standards among the leading contributing factors [8]. Taken together, these findings point toward a shared underlying deficit: insufficient translation of legal knowledge into stable, situation-appropriate professional conduct.

Despite growing recognition of this issue, the concept of legal literacy in clinical nurses remains insufficiently defined. Existing studies use terms such as legal knowledge, legal awareness, legal cognition, legal liability knowledge, legal-ethical competence, and law-based practice capacity in inconsistent and sometimes interchangeable ways [9,10,11,12,13]. As a result, measurement tools and educational interventions have operationalized this domain heterogeneously. Some instruments equate legal literacy with factual knowledge of laws and regulations [14,15], whereas others include awareness or attitudinal dimensions without clearly distinguishing risk perception, responsibility orientation, compliance intention, and practical legal reasoning [16]. More importantly, many existing tools do not adequately capture nurses’ ability to identify legal issues, reason through normative obligations, and implement lawful action in specific clinical situations [17]. This conceptual fragmentation limits construct validity, weakens comparability across studies, and reduces the precision of educational and management interventions [18].

A further challenge is that legal literacy is conceptually close to, but not identical with, several adjacent constructs. Legal knowledge, legal awareness, legal cognition, and medical ethics each reflect important aspects of the normative context of nursing practice, but none fully captures the integrated capacity required for legally accountable clinical action. Clarifying the relationships among these constructs is therefore essential for developing valid measurement instruments, designing targeted legal education, and strengthening nursing management strategies.

Concept analysis provides a systematic approach for addressing such conceptual ambiguity. By examining how a concept is used across the literature, identifying defining attributes, constructing illustrative cases, and delineating antecedents, consequences, and empirical referents, concept analysis can generate a clearer operational structure for future research and practice [19,20]. Walker and Avant’s method offers a structured eight-step framework that is particularly suitable for clarifying complex and context-dependent nursing concepts [21]. Legal literacy in clinical nurses is such a concept, as it is shaped by evolving legislation, institutional governance, healthcare delivery models, and nurses’ professional development.

Accordingly, the purpose of this study was to conduct a concept analysis of legal literacy in clinical nurses using Walker and Avant’s method, with the aims of clarifying its conceptual boundaries, identifying its defining attributes, constructing illustrative cases, delineating its antecedents and consequences, identifying its empirical referents, and formulating an operational definition. The findings are intended to provide a conceptual and structural foundation for the development of valid measurement instruments and the design of targeted educational and management strategies aimed at strengthening nurses’ legal competence in clinical practice.

## 2. Methods

### 2.1. Study Design

This study used Walker and Avant’s concept analysis method to clarify the meaning, defining attributes, antecedents, consequences, and empirical referents of legal literacy in clinical nursing practice [21]. Walker and Avant’s approach was selected because it provides a structured process for examining concepts that are frequently used but not yet clearly defined in professional practice [21].

The analysis followed the eight steps proposed by Walker and Avant: selecting the concept, determining the aims of the analysis, identifying uses of the concept, determining defining attributes, constructing a model case, identifying additional cases, identifying antecedents and consequences, and defining empirical referents. In the present study, “legal literacy in clinical nurses” was selected as the target concept. The purpose of the analysis was to clarify its conceptual boundaries and to distinguish it from adjacent concepts, including legal knowledge, legal awareness, legal cognition, and medical ethics. Literature from nursing, healthcare, law, public health, social sciences, and ethics was reviewed to identify the use of the concept and to support the development of its defining attributes.

### 2.2. Literature Search Strategy

The literature search was conducted to support concept analysis rather than to perform a systematic review or meta-analysis. Therefore, the purpose of the search was not to estimate effect sizes or synthesize intervention outcomes, but to identify how legal literacy and related concepts have been defined, used, measured, and distinguished in nursing and healthcare practice.

The search was conducted in two stages. The first stage was the primary search, which aimed to identify sources directly relevant to legal literacy and closely related legal competencies in nursing and healthcare contexts. The second stage was a supplementary conceptual search, which was conducted to clarify the boundary between legal awareness and legal consciousness.

#### 2.2.1. Primary Search

The primary search was conducted in Wanfang, VIP, CNKI, CINAHL, Cochrane Library, Embase, PubMed and Web of Science from inception to December 2024. Search terms were developed around three domains: the target concept, adjacent legal concepts, and the clinical nursing context.

The search terms included: “legal literacy,” “legal knowledge,” “legal awareness,” “legal cognition,” “legal liability,” “medical law,” “health law,” “nursing law,” “legal responsibility,” “professional responsibility,” “nurse*,” “nursing,” “clinical nurse*,” “healthcare professional*,” and “clinical practice.”

A representative search syntax was as follows:

(“legal literacy” OR “legal knowledge” OR “legal awareness” OR “legal cognition” OR “legal liability” OR “medical law” OR “health law” OR “nursing law” OR “legal responsibility” OR “professional responsibility”)

AND

(“nurse*” OR “nursing” OR “clinical nurse*” OR “healthcare professional*” OR “clinical practice”)

Database-specific search strategies were adapted according to the indexing rules and search functions of each database, including differences in title/abstract fields, subject headings, keywords, and Chinese search terms. The complete database-specific search strategies are provided in Supplementary Table S1.

#### 2.2.2. Supplementary Conceptual Search

In addition to the primary search, a supplementary conceptual search was conducted using the term “legal consciousness.” This was added because legal consciousness is a broader socio-legal construct closely related to legal awareness, and it was relevant to clarifying the conceptual boundary between legal awareness and legal consciousness.

The supplementary search was not intended to broaden the primary empirical source pool or to redefine the scope of the concept analysis. Instead, it was used to verify whether additional socio-legal literature was necessary to support the distinction between legal awareness and legal consciousness. The supplementary search did not identify any additional sources that met the formal eligibility criteria for inclusion in the primary concept analysis. Therefore, the number of sources included in the primary analysis remained unchanged at 56. However, the search informed the refinement of the conceptual discussion in Section 3.1.2, particularly the distinction between legal awareness as a professional healthcare-related construct and legal consciousness as a broader socio-legal concept.

### 2.3. Eligibility Criteria

Sources were included if they met one or more of the following criteria:

(1) they provided definitions, descriptions, or theoretical discussions of legal literacy or closely related legal concepts;
(2) they examined legal knowledge, legal awareness, legal cognition, legal liability, legal responsibility, or legal and ethical responsibilities among nurses or other healthcare professionals;
(3) they developed or used instruments measuring legal knowledge, legal awareness, legal liability, or legally relevant professional competencies;
(4) they contributed to identifying the attributes, antecedents, consequences, or empirical referents of legal literacy in clinical nursing practice;
(5) they helped distinguish legal literacy from adjacent concepts such as legal knowledge, legal awareness, legal cognition, legal consciousness, medical ethics, and professional responsibility.

Sources were excluded if they focused only on legal doctrine without relevance to healthcare or nursing practice, discussed patient legal rights without reference to healthcare professionals’ responsibilities or competencies, did not contribute to conceptual clarification, were duplicates, or had unavailable full text. Sources that discussed legal consciousness only in a broad socio-legal or political context without relevance to healthcare professional practice were not included in the primary concept analysis.

### 2.4. Source Selection and Analysis

**Figure 1:**
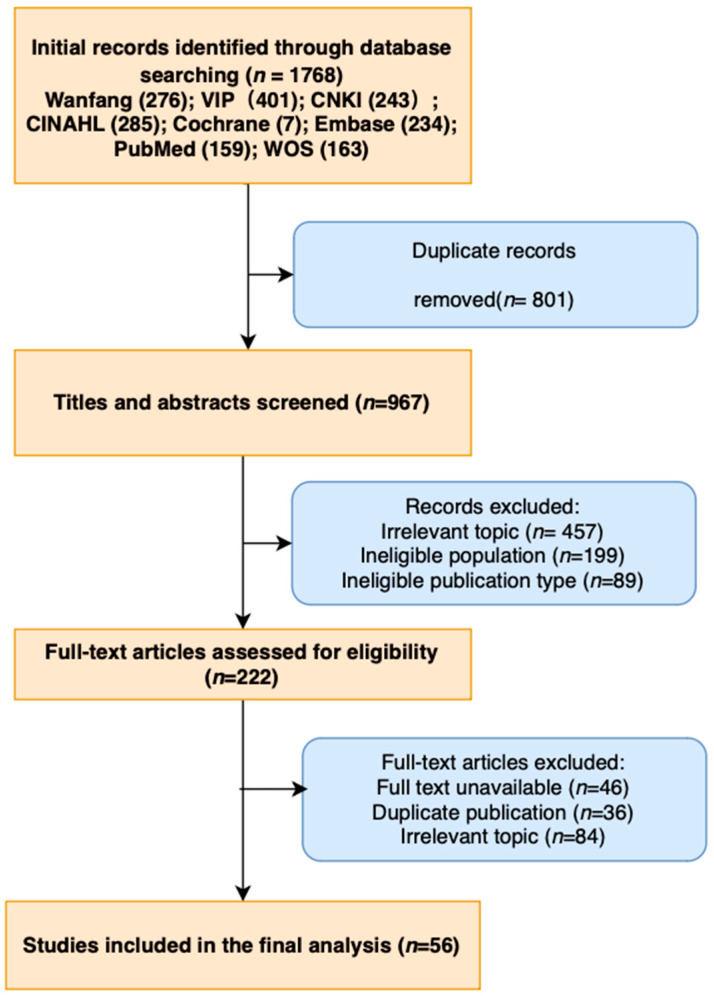
Literature screening process and results for the concept analysis.

Source selection, data extraction, and content analysis were conducted in a structured and iterative manner. The database search initially identified 1768 records, including records from Wanfang (*n* = 276), VIP (*n* = 401), CNKI (*n* = 243), CINAHL (*n* = 285), Cochrane Library (*n* = 7), Embase (*n* = 234), PubMed (*n* = 159), and Web of Science (*n* = 163). After removing 801 duplicate records, 967 records remained for title and abstract screening. During this stage, 745 records were excluded because they were irrelevant to the topic (*n* = 457), from ineligible populations (*n* = 199), or from ineligible publication types (*n* = 89). The full texts of 222 articles were then assessed for eligibility. Of these, 166 articles were excluded because the full text was unavailable (*n* = 46), the article was a duplicate publication (*n* = 36), or the topic was irrelevant to the purpose of concept analysis (*n* = 84). Finally, 56 sources were included in the concept analysis. A PRISMA-style flow diagram was used to present the identification, screening, eligibility assessment, and inclusion process (Figure 1), although this study was conducted as a concept analysis rather than a systematic review [22].

The supplementary conceptual search using “legal consciousness” did not identify any additional sources that met the formal eligibility criteria for inclusion in the primary concept analysis. Therefore, the number of sources included in the primary analysis remained unchanged at 56. A detailed summary of the included sources and their relevance to the identification of defining attributes, antecedents, consequences, empirical referents, and conceptual boundaries is provided in Supplementary Table S2. Reference management and duplicate removal were performed using Zotero (version 6.0.37; Corporation for Digital Scholarship, Vienna, VA, USA; https://www.zotero.org/). Data extraction, coding records, and tabulation were conducted manually using Microsoft Word for Mac (version 16.53 (21091200); Microsoft Corporation, Redmond, WA, USA) and Microsoft Excel for Mac (version 16.53 (21091200); Microsoft Corporation, Redmond, WA, USA).

Data were extracted using a standardized form developed by the research team. Extracted information included author, year, country or region, disciplinary field, study type, definitions or descriptions of legal literacy and related concepts, conceptual dimensions, measurement tools, antecedents, consequences, empirical referents, and examples of legally relevant clinical practice.

Qualitative content analysis was used to identify conceptual meanings and defining attributes. The unit of analysis was a meaning unit related to the definition, use, attribute, antecedent, consequence, or empirical referent of legal literacy and adjacent concepts. Two reviewers independently coded the extracted meaning units. Similar codes were compared and grouped into preliminary categories, which were refined through constant comparison across theoretical, empirical, and measurement-based sources. The final categories were then mapped onto Walker and Avant’s analytical components, including defining attributes, antecedents, consequences, and empirical referents.

Analytic trustworthiness was enhanced through independent dual-reviewer coding, comparison of coding results, team discussion, repeated checking of categories against the original texts, and maintenance of an audit trail, including search records, screening decisions, extraction forms, coding notes, and category refinement records. Disagreements in screening, coding, or category development were resolved through discussion or, when necessary, adjudicated by a third reviewer.

### 2.5. Quality Appraisal and Conceptual Relevance Assessment

A formal methodological quality appraisal was not conducted because this study was a concept analysis rather than an evidence synthesis. The included sources were methodologically heterogeneous, including conceptual papers, theoretical discussions, empirical studies, instrument-development studies, legal literature, ethical literature, and nursing practice-related studies. Therefore, applying a single formal quality appraisal tool was not appropriate. Instead, each source was assessed for conceptual relevance and contribution to the analysis, including whether it helped define legal literacy or related concepts, clarify conceptual boundaries, identify defining attributes, antecedents, consequences, or empirical referents, or illustrate the application of legal literacy in clinical nursing practice. No source was excluded solely on the basis of methodological quality; instead, inclusion was determined by conceptual relevance and contribution to the aims of the concept analysis.

## 3. Results

### 3.1. Distinguishing Related Concepts

Within Walker and Avant’s framework, distinguishing a target concept from adjacent and potentially overlapping concepts is an essential preliminary step, because it clarifies the conceptual boundaries that the defining attributes must subsequently delimit [21]. In the nursing literature on legal issues, four concepts are frequently used in ways that overlap with, or are sometimes conflated with, legal literacy: legal knowledge, legal awareness, legal cognition, and medical ethics [2,12,23]. In this analysis, these concepts are not treated as entirely unrelated constructs. Rather, they are positioned along a conceptual continuum from factual knowledge, to subjective interpretation and value orientation, and finally to situated professional action. Clarifying these relationships helps specify what is distinctive about legal literacy in clinical nursing practice.

#### 3.1.1. Legal Knowledge

Legal knowledge refers to factual mastery of legal provisions, institutional rules, types of liability, and procedural requirements, and is commonly measured using objective knowledge tests or questionnaires [24]. In nursing research, legal knowledge assessments typically cover nurses’ rights and obligations in practice, patients’ rights, documentation standards, and the boundaries of clinical responsibility. For example, Ibrahim et al. [13] developed a 63-item questionnaire assssing thirteen dimensions of legal liability—including negligence, malpractice, informed consent, invasion of privacy, and order execution—all of which are constituted by factual legal content. Similarly, Kumar et al. [25] assessed nurses’ knowledge of legal and ethical responsibilities in psychiatric nursing using a structured knowledge questionnaire scoring responses at three levels. Across these instruments, the consistent operationalization is factual recall or recognition of legally relevant information. Legal knowledge is thus a necessary cognitive foundation of legal literacy but does not itself encompass the value orientations or practical capacities that distinguish the fuller construct.

#### 3.1.2. Legal Awareness

Legal awareness refers to healthcare professionals’ cognition, attitudes, evaluations, and affective orientation toward medical law, health regulations, institutional rules, and the normative order governing clinical practice [10]. This construct extends beyond factual knowledge. Its cognitive dimension concerns whether nurses recognize the relevance of law to clinical practice; its attitudinal dimension concerns whether they accept legal rules and procedures as professionally important; its evaluative dimension concerns how they judge the legitimacy, necessity, and practical significance of legal requirements; and its affective orientation concerns their respect for, trust in, or concern about law and legal accountability in care situations.

Legal awareness is closely related to, but should not be used interchangeably with, legal consciousness. In socio-legal scholarship, legal consciousness is a broader concept that concerns how individuals understand, experience, interpret, and position law in everyday social life [26]. By contrast, legal awareness in the healthcare context is used here in a narrower professional sense, referring to nurses’ awareness of legal norms and their subjective endorsement of lawful practice, patient rights, professional responsibility, and procedural compliance [27]. This distinction is important because legal consciousness provides a broader socio-legal perspective, whereas legal awareness in this study is more directly tied to professional practice and legal education in clinical nursing.

In nursing research, Kim et al. [16] developed a 25-item Legal Awareness Questionnaire to measure nurses’ subjective perceptions of legal liability in patient assessment and communication. Their findings suggested that legal awareness alone did not necessarily predict liability-related attitudes, indicating that legal education should not only transmit legal information but also address values, attitudes, and professional responsibility. It should also be noted that the term “legal awareness” has not always been used rigorously in nursing studies. Some studies describe their outcome as legal awareness, while the items used are in fact objective knowledge questions that assess factual recall rather than attitudinal endorsement of lawful practice [25].

For the present concept analysis, legal awareness is therefore understood as nurses’ subjective recognition and endorsement of lawful practice, protection of patient rights, assumption of professional responsibility, and compliance with clinical procedures, rather than as legal knowledge per se. Compared with legal literacy, legal awareness constitutes an important value-attitudinal component, but it remains insufficient on its own. Legal literacy additionally requires systematic normative understanding and the capacity to translate legal and professional norms into contextually appropriate clinical action.

#### 3.1.3. Legal Cognition

Legal cognition refers to nurses’ understanding and subjective interpretation of the meaning, relevance, and significance of legal norms in clinical practice [12,28]. It is broader than legal knowledge because it not only involves knowing legal provisions, but also involves interpreting why those provisions matter and how they relate to professional responsibility. At the same time, it is narrower than legal literacy because it does not necessarily include stable value internalization or the ability to enact lawful practice in complex clinical situations.

Conceptually, legal cognition occupies an intermediate position between legal knowledge and legal literacy. Legal knowledge mainly concerns factual recognition of rules, whereas legal cognition concerns nurses’ comprehension and appraisal of the role of law in professional practice. For example, a nurse may know that informed consent is legally required, and may also understand that this requirement is related to patient autonomy, risk communication, and institutional accountability. This reflects legal cognition. However, unless this understanding is further internalized as a professional value and translated into appropriate action in concrete clinical encounters, it remains insufficient to constitute full legal literacy.

Legal cognition also differs from legal awareness. Legal cognition emphasizes understanding and interpretation, whereas legal awareness places greater emphasis on subjective endorsement, attitudes, evaluations, and affective orientation toward law. In other words, legal cognition concerns how nurses understand the legal significance of clinical situations, while legal awareness concerns whether they value, accept, and orient themselves toward lawful and responsible practice. Legal literacy incorporates both dimensions, but further requires the practical ability to act lawfully and responsibly in specific clinical contexts.

#### 3.1.4. Medical Ethics

Medical ethics refers to the value principles and moral norms that govern relationships among persons, professions, and society in healthcare [29,30]. In its classic formulation, the four-principles framework proposed by Beauchamp and Childress, namely respect for autonomy, non-maleficence, beneficence, and justice [31], is widely applied to clinical ethical reasoning and the analysis of moral dilemmas. Nursing codes of ethics further operationalize these principles into concrete professional obligations, including informed consent, privacy and confidentiality, and respect for patients’ treatment decisions [3,32].

Medical ethics is closely related to legal literacy in clinical nurses, but the two are not interchangeable. Law establishes formally accountable normative boundaries and emphasizes rights, duties, procedures, evidence, and responsibility. Ethics provides moral justification and value-based reasoning and emphasizes broader aspirational standards, such as beneficence, dignity, compassion, fairness, and respect for persons [33,34]. The two domains often overlap in clinical practice, but they differ in normative source, reasoning logic, and accountability mechanism.

Informed consent provides a useful example. Ethically, informed consent reflects respect for patient autonomy and requires meaningful communication, adequate disclosure, and voluntary decision-making. Legally, informed consent also requires procedural validity, competent authorization, appropriate documentation, and evidence that the required process has been followed [35]. Thus, a nurse may have strong ethical concern for patient autonomy but still lack specific knowledge of legal procedures, documentation requirements, or institutional accountability. Plaiasu et al. [35] similarly demonstrated that healthcare professionals may show gaps in legal knowledge regarding informed consent and confidentiality despite recognizing their ethical importance. Podgorica et al. [36] also found that identifying ethical and legal issues in elder care involved distinct forms of reasoning, with legal issues requiring more context-specific normative knowledge.

Therefore, legal literacy in clinical nurses is more centrally concerned with understanding legal norms, recognizing rights and responsibilities, and enacting lawful practice in clinical situations. Medical ethics is more centrally concerned with moral judgment, value-based reasoning, and the resolution of ethical dilemmas in care. Although both are necessary for professional nursing practice, legal literacy emphasizes legally accountable practice, whereas medical ethics emphasizes morally justified practice.

### 3.2. Defining Attributes of Legal Literacy in Clinical Nurses

Defining attributes are the core of concept analysis: they are the characteristics that appear repeatedly across multiple uses of a concept and most reliably distinguish it from related concepts [21]. The aim is not to identify the maximum number of attributes, but to derive the smallest set of highly discriminative features that are both necessary and sufficient to constitute the concept. Care was also taken to distinguish attributes from antecedents, consequences, and empirical referents, which are functionally distinct elements in Walker and Avant’s framework.

Systematic comparison of the included literature revealed that, despite the heterogeneity of terms used, including legal knowledge, legal awareness, legal cognition, knowledge of legal liability, and legal-ethical competence the content of these studies converged repeatedly on three features: (1) understanding of legal norms and institutional requirements relevant to nursing; (2) a stable orientation toward protecting patients’ rights and bearing professional responsibility; and (3) a tendency to make lawful judgments and enact normative action in concrete nursing situations. These three features were consistently present across sources from nursing, law, and health sciences, and were found to differentiate legal literacy from the four adjacent concepts examined in Section 3.1. Accordingly, the defining attributes of legal literacy in clinical nurses are identified as: normative understanding, value internalization oriented toward rights and responsibilities, and law-based situational practice.

#### 3.2.1. Normative Understanding

Normative understanding refers to the capacity of clinical nurses to comprehend the laws, regulations, institutional rules, procedural requirements, and applicable boundaries relevant to nursing practice, and on this basis to form a functional framework for judging the legality and propriety of nursing conduct. It does not require rote memorization of legal text, but an understanding of what legal norms mean, where they apply, and how they relate to nursing decisions and actions.

This attribute is the most consistently documented across the nursing literature on legal issues. Studies focusing on nurses’ mastery of legal regulations and core legal issues—including order execution, informed consent, privacy protection, nursing documentation, duty of care, adverse-event management, and dispute handling—share the common object of assessing whether nurses can identify and understand the legal norms relevant to their practice [13,17,25,35,37]. Wang et al. [12] found that clinical nurses scored relatively well on order execution and legal documentation standards, but substantially less well on privacy rights, informed consent, and duty-of-care boundaries—illustrating both the discriminative validity of this attribute and the heterogeneity of normative understanding across domains. Internationally, Ibrahim et al. [13] similarly found that the majority of Egyptian clinical nurses demonstrated poor to moderate levels of knowledge across their 13 legal liability dimensions, with particularly notable gaps in assault, false imprisonment, and physician order execution. Normative understanding constitutes a defining attribute because it provides the irreducible cognitive basis for legal identification, boundary-setting, and subsequent judgment: without it, legal literacy in clinical nurses would have no substantive foundation.

#### 3.2.2. Value Internalization Oriented Toward Rights and Responsibilities

Value internalization refers to the transformation of legal requirements—concerning patient-rights protection, lawful practice, professional responsibility, and safeguarding of nurses’ own rights—into a stable professional value orientation that functions as an active basis for nursing judgment, rather than merely as an external constraint. This attribute distinguishes legal literacy from the possession of legal knowledge alone, because it concerns whether nurses genuinely endorse and are oriented toward acting lawfully, rather than whether they can recall legal information.

Although nursing studies have rarely rigorously separated legal awareness, legal cognition, and legal attitudes as constructs, the substantive literature consistently demonstrates that nurses’ engagement with legal issues involves normative endorsement as well as factual knowledge. Research on informed consent, privacy protection, and patient-rights protection essentially concerns whether nurses regard rights protection as an operative basis for nursing decision-making in their everyday practice [10,11,28,38,39,40]. Research on legal liability, professional boundaries, and risk prevention reflects whether nurses proactively orient toward procedural compliance and responsibility-bearing as professional values, not merely as externally imposed rules. A Korean study by Kim et al. [16] found no significant correlation between legal awareness scores and liability attitudes, and concluded that legal education requires a specific focus on attitude-oriented and values-oriented dimensions rather than knowledge delivery alone—directly evidencing that value internalization constitutes a functionally distinct attribute that is not automatically produced by normative understanding. International professional competency frameworks further reflect this, incorporating legal and ethical accountability as a required professional value for advanced practice nurses within national nursing competency standards [41]. Value internalization therefore represents the mediating structure through which normative understanding is transformed from recalled knowledge into stable professional orientation, and without this attribute, legal literacy would not be reliably expressed in practice.

#### 3.2.3. Law-Based Situational Practice

Law-based situational practice refers to the capacity of clinical nurses to identify legal issues, reason about legal boundaries, select normatively appropriate courses of action, and implement compliant conduct in the specific clinical situations they encounter. This attribute is the most distinctively occupational of the three and most clearly differentiates legal literacy in clinical nurses from legal literacy in the general population, where practical translation into a professional context of action is not required.

Clinical nursing does not engage with law in the abstract. Legal norms intersect with nursing practice across a continuous cycle of assessment, procedure, documentation, communication, handover, and adverse-event management [42]. Studies on order execution, nursing documentation standards, emergency care obligations, restraint and protection procedures, and dispute-handling practices all address, at least implicitly, how nurses make decisions that are lawful, traceable, and defensible in specific situations [43,44]. The web-based educational program developed by Kim [45] for malpractice prevention was grounded precisely in this practical-situational dimension, using scenario-based learning to develop nurses’ capacity to identify liability-relevant situations and respond appropriately. Internationally, Hasan et al. [9] assessed emergency nurses’ knowledge and attitudes toward work-related legal issues using a context-specific, scenario-informed instrument, confirming that legal competence in clinical settings cannot be fully captured by generic knowledge measures. Wang et al. [38] further found that clinical nurses in China identified the translation of legal requirements into specific practice behaviors, particularly in communication, documentation, and adverse-event management as the most practically challenging dimension of legal competence. Law-based situational practice is thus a defining attribute because it reflects that legal literacy in clinical nurses is not a static knowledge store nor an abstract disposition, but a context-specific occupational capacity that must be expressed in the concrete situations of nursing practice to carry practical meaning.

In summary, the defining attributes of legal literacy in clinical nurses are normative understanding, value internalization oriented toward rights and responsibilities, and law-based situational practice. These attributes are not isolated, but form an internalization chain moving from cognition to orientation to action: normative understanding provides the cognitive basis for recognizing legal issues; value internalization represents the professional assimilation of legal requirements into a stable normative orientation; and law-based situational practice enables the compliant judgment and lawful conduct that constitute legal literacy’s expression in clinical reality.

### 3.3. Cases

Cases serve to test the proposed defining attributes by illustrating combinations of their presence and absence across plausible clinical scenarios [21]. A model case contains all defining attributes; a borderline case contains some but not all; a related case involves a concept that is closely adjacent to but genuinely distinct from the target concept; and a contrary case illustrates the absence of all defining attributes.

#### 3.3.1. Model Case

Nurse A in a hospital ward is preparing to perform an invasive nursing procedure. She first checks the medical order and verifies the patient’s identity, and assesses the patient’s condition and clinical indication. She explains the purpose, process, potential risks, alternatives, and precautions in language the patient can understand, confirms understanding, and obtains valid informed consent before proceeding. During the procedure, she adheres to technical specifications and institutional protocols, protects the patient’s privacy and safety, monitors responses, and addresses any abnormalities promptly. After the procedure, she completes nursing documentation in a timely, objective, and complete manner. When the patient and family raise questions about the necessity and risk of the intervention, Nurse A communicates professionally, explains the applicable legal provisions, nursing standards, and institutional rules, and delineates the patient’s rights to information, choice, and privacy. When necessary, she collaborates with the responsible physician and nurse manager to resolve the issue, thereby safeguarding both the patient’s legal rights and her own occupational security.

This case demonstrates all three defining attributes of legal literacy in clinical nurses: normative understanding of relevant procedural requirements and legal provisions, value internalization in the form of patient-rights protection as an operative basis for decision-making, and law-based situational practice manifested in lawful, procedurally defensible, and communicatively compliant conduct throughout the care process. It therefore constitutes a model case of legal literacy in clinical nurses.

#### 3.3.2. Borderline Case

Nurse B, working in a hospital ward, consistently checks medical orders and patient identity, performs procedures according to nursing standards, and completes documentation accurately. When patients ask questions, Nurse B provides professional and clear responses and reminds patients of necessary precautions. These behaviors demonstrate normative understanding and some elements of law-based situational practice. However, after shift handover, while discussing a patient’s care priorities with a colleague in a shared corridor, Nurse B directly mentions the patient’s sensitive diagnosis and family-related private information within earshot of other patients and families. The patient subsequently complains of a privacy violation, and following investigation the hospital requires Nurse B to undertake additional privacy-protection training and records an internal disciplinary notation.

In this case, Nurse B possesses adequate legal knowledge and demonstrates compliant performance across most clinical procedures, but has not fully internalized privacy protection as a stable professional orientation that operates across all care contexts. Value internalization is therefore incompletely present: knowledge has not been assimilated into a consistently enacted normative orientation. This case is borderline rather than a full instance of legal literacy.

#### 3.3.3. Related Case

A related case contains a concept closely associated with the target concept but lacking one or more of its defining attributes and therefore not constituting an instance of legal literacy itself [21]. For legal literacy in clinical nurses, ethical competence constitutes such a related concept.

Nurse D is a senior nurse in a palliative care ward with extensive experience in care of patients at end of life. When a terminally ill patient requests that painful and burdensome treatment be withdrawn, Nurse D recognizes the ethical significance of the situation and proactively coordinates a family meeting with the attending physician and social worker to ensure that the patient’s wishes are heard and respected. Drawing on her understanding of patient autonomy, non-maleficence, and beneficence, she advocates sensitively for the patient’s right to make treatment decisions consistent with their values, and ensures that all discussions are conducted with compassion. Her colleagues and the family consistently describe her as a nurse of exceptional moral integrity.

However, when documentation of the withdrawal decision is prepared, Nurse D is uncertain about the specific legal procedural requirements: she is unsure which forms require physician co-signature under the applicable regulations, whether the patient’s written refusal of further intervention constitutes a valid legal record under institutional policy, and what the documentation requirements are for notifying the clinical nurse manager. She defers these decisions to the physician without independently verifying the applicable standards and does not document her own nursing assessment of the patient’s decision-making capacity. When an administrator later reviews the case, procedural gaps in the nursing record are identified.

This case illustrates ethical competence—including genuine respect for patient autonomy, skillful interprofessional advocacy, and strong moral sensitivity—without the normative understanding and law-based situational practice that together constitute legal literacy in clinical nurses. As Podgorica et al. [36] found, ethical and legal dimensions of clinical decision-making, although related, require distinct forms of knowledge and competence; a nurse may demonstrate strong ethical reasoning without reliably identifying or applying the specific legal and procedural requirements that govern the same situation. This related case therefore clarifies that ethical competence and legal literacy are adjacent and mutually reinforcing constructs, but neither is a component nor a substitute for the other.

#### 3.3.4. Contrary Case

Nurse C in a ward starts an invasive procedure without verifying the patient’s identity or reviewing the medical order. She does not explain the purpose, risks, or precautions of the procedure to the patient and does not obtain informed consent. During the procedure, she fails to apply aseptic principles and technical standards and omits key procedural steps. Afterward, she does not document the procedure in a timely or complete manner; some records are subsequently completed retrospectively, with inconsistent times and content. When the patient develops discomfort and raises concerns, Nurse C dismisses these, stating that the procedure is routine, and declines to provide any explanation of its clinical or legal basis. When the family requests relevant documentation and an account of the intervention, she responds with emotional argument and implies that pursuing a complaint would create difficulties for the patient. She neither manages the situation in accordance with law and institutional policy nor activates the hospital’s incident-reporting process or escalates to her nurse manager. The patient subsequently files a formal complaint, and investigation identifies clear violations across identity verification, disclosure and consent, standardized practice, documentation standards, and dispute-handling procedures.

This case illustrates the absence of all three defining attributes of legal literacy in clinical nurses: lack of normative understanding of relevant procedural and legal requirements, lack of value internalization with respect to patient-rights protection and professional responsibility, and lack of law-based situational practice, manifested in non-compliant conduct, falsified records, and legally inappropriate communication throughout the care process. It therefore constitutes a contrary case of severely deficient legal literacy.

### 3.4. Antecedents of Legal Literacy in Clinical Nurses

Antecedents are events, conditions, or circumstances that must precede or accompany the emergence of a concept and function as its necessary contextual prerequisites [21]. Based on the included literature and relevant professional documents, the antecedents of legal literacy in clinical nurses are organized at three levels.

#### 3.4.1. Macro-Level: Legal Environment and Sectoral Governance

The continuing development of the healthcare legal environment, including the enactment and ongoing refinement of nursing legislation, patient-rights protections, documentation requirements, and dispute-handling regulations, provides both the institutional foundation and the normative impetus for the development of legal literacy in clinical nurses [46,47]. As legal provisions become more explicit, the normative demands on clinical nursing practice intensify, and the boundaries of rights, obligations, and accountability become more clearly specified. Wang et al. [12] found that Chinese clinical nurses identified clear legal provisions and enabling legislation as essential preconditions for understanding and fulfilling their legal roles, and that ambiguity in legal frameworks created significant practice uncertainty. This macro-level structural condition thus constitutes a prerequisite for the emergence and consolidation of normative understanding as a defining attribute.

#### 3.4.2. Meso-Level: Organizational Risk Governance and Compliance Systems

The organizational context of healthcare institutions, including the quality and accessibility of legal management systems, adverse-event reporting structures, risk governance processes, and the availability of institutional legal and managerial support directly mediates the extent to which nurses can translate legal knowledge and awareness into stable patterns of law-based practice [48]. Clinical nursing occurs in settings with substantial legal exposure: informed disclosure obligations, privacy protection requirements, documentation standards, and dispute-management protocols all generate ongoing compliance demands. Organizational systems that provide executable standards, continuing training, supervision, and accessible consultation are critical for enabling the internalization and situational application of legal norms [49,50]. Conversely, organizations characterized by blame-oriented cultures or inadequate risk infrastructure may actively impede the development of legal literacy, as nurses in such environments have been shown to withhold incident reports and avoid legally appropriate disclosure out of fear of punitive consequences [51].

#### 3.4.3. Micro-Level: Individual Professional Experience and Self-Efficacy

Individual-level antecedents include years of nursing experience, specialty and exposure to high-risk situations, prior encounters with disputes or adverse events, personal learning experiences and legal education, and motivation and self-efficacy in relation to legal issues [45,52,53,54,55]. Multiple studies have found that more experienced nurses and those with higher education levels demonstrate relatively stronger legal knowledge and awareness, although some research also documents that recently graduated nurses may demonstrate greater legal awareness due to more recent and intensive legal education during training [54]. These individual-level factors shape nurses’ sensitivity to legal issues, their capacity for self-directed updating of legal knowledge, and their sustained motivation for law-based practice.

Taken together, these three levels of antecedents constitute the institutional context, organizational support conditions, and individual developmental basis that together enable the emergence of legal literacy’s defining attributes in clinical nurses, and provide a plausible precondition for the stable appearance of its defining attributes.

### 3.5. Consequences of Legal Literacy in Clinical Nurses

Consequences are the phenomena or outcomes that follow from the concept’s presence and can be regarded as its direct or indirect effects [21]. Based on the included literature, the consequences of legal literacy in clinical nurses are evident at three levels.

#### 3.5.1. Individual Level: Enhanced Professional Competence and Risk Management Capacity

Nurses with higher legal literacy demonstrate greater capacity to identify liability-relevant situations, maintain appropriate procedural standards, and manage risk-prone interactions in ways that reduce the probability of adverse events and professional liability exposure [45]. Clinical nurses with stronger legal literacy are better positioned to adhere to normative standards in assessment, procedures, documentation, communication, and privacy protection, thereby identifying dispute-precipitating factors earlier and managing them through compliant practice [56]. Legal literacy also contributes to professional development: nurses with stronger legal competence demonstrate greater professional confidence, more prudent decision-making in complex situations, and stronger accountability orientation—qualities that contribute to advancement in management, specialist practice, and quality oversight roles [12,57].

#### 3.5.2. Organizational Level: Reduced Dispute Risk and Improved Care Quality

When nurses collectively demonstrate adequate legal literacy, the implementation of core care systems is more likely to move beyond formal compliance into concrete procedural behavior across assessment, documentation, reporting, and communication, thereby improving process quality and reducing omissions [58]. Legal literacy contributes to the reduction of disputes at source: when nurses fulfill disclosure obligations accurately and respect patients’ rights, informational asymmetries that commonly precipitate complaints and formal disputes are reduced [59]. When disputes do arise, nurses with stronger legal literacy can participate lawfully and constructively in resolution processes, contributing to outcomes that protect both patient rights and institutional accountability [60]. At the organizational level, the consequence is not merely fewer individual nursing errors, but greater normativity, traceability, and evaluability across nursing practice systems.

#### 3.5.3. Patient-Rights Level: Protection of Safety and Lawful Interests

The ultimate consequence of legal literacy in clinical nurses is expressed in the safety and protection of patients’ lawful rights [56]. Nurses who practice in accordance with legal and institutional standards are better able to implement key procedural safeguards, such as systematic observation, double-checking, documentation, and timely escalation of abnormal findings, which may help reduce the risks associated with procedural omissions and incomplete records. Such nurses are also more likely to implement informed disclosure, privacy protection, and dignity-preserving communication accurately, thereby reducing harm to patients’ rights caused by insufficient awareness or normative uncertainty. Legal literacy therefore affects not only the normativity of nursing conduct but also the safety and legal legitimacy of the care experience received by patients.

### 3.6. Empirical Referents of Legal Literacy in Clinical Nurses

Empirical referents are observable or measurable phenomena that indicate the presence of a concept and its defining attributes in practice, and provide the operational basis for instrument development [21]. A critical function of this step is to evaluate the extent to which existing measurement approaches correspond to the concept’s defining attributes, thereby identifying gaps that justify new instrument development.

#### 3.6.1. Existing Instruments and Their Attribute Coverage

A systematic review of measurement approaches across the included literature identified three representative categories of instrument, each of which covers one or two of the defining attributes but not all three.

Instruments predominantly targeting normative understanding. The most numerous category, these tools assess factual legal knowledge through objective questionnaires, knowledge tests, or structured knowledge checklists. Ibrahim et al. [13] developed a 63-item questionnaire for Egyptian clinical nurses to assess 13 dimensions of legal liability, including negligence, malpractice, informed consent, and invasion of privacy, using case-based, multiple-choice, and true-false items. Kumar et al. [25] assessed knowledge of legal and ethical responsibilities in psychiatric nursing using a structured questionnaire scored at three levels. Wang et al. [12] surveyed Chinese clinical nurses’ legal education and knowledge across dimensions including patient rights, documentation standards, and order execution. While these instruments provide systematic coverage of the normative understanding attribute, they do not assess value orientation or situational judgment capacity, and their dimensional structures were not derived from a theoretically grounded conceptual framework for legal literacy, limiting their construct validity.

Instruments targeting legal awareness and attitudes alongside knowledge. Kim et al. [16] developed a 25-item Legal Awareness Questionnaire for Korean nurses, paired with an Attitude toward Duty and Liability Questionnaire, to assess both knowledge and attitudinal dimensions of legal liability in communication and assessment contexts. This approach is closer to capturing both normative understanding and elements of value internalization; however, the Legal Awareness Questionnaire was found to measure primarily factual recall rather than the attitudinal endorsement of lawful practice, illustrating the conceptual conflation problem identified in Section 3.1.2. Hasan et al. [9] assessed both knowledge and attitudes of emergency nurses toward work-related legal issues in a cross-sectional study, using items addressing legal scenarios encountered in emergency practice; this tool partially captures law-based situational practice through its scenario-based items but was designed as a standalone survey instrument rather than a validated scale with confirmed factor structure.

Web-based and scenario-based educational evaluation tools. Kim [45] developed a web-based diagnostic evaluation program for malpractice prevention, using scenario-based learning and self-assessment items grounded in actual malpractice cases to evaluate nurses’ identification of liability risks and selection of appropriate responses. This approach most closely approximates the law-based situational practice attribute by embedding legal reasoning in realistic clinical scenarios. However, it was designed primarily as an educational and diagnostic tool rather than a psychometrically validated assessment instrument, limiting its utility as a measurement instrument for research purposes.

#### 3.6.2. Gaps in Existing Measurement Approaches

Taken together, the existing instruments reveal a consistent pattern of partial and asymmetric coverage relative to the three defining attributes identified in this analysis. Normative understanding is the best-measured attribute, with multiple instruments providing broad factual knowledge coverage; however, most lack systematic dimensional construction grounded in a theoretically derived conceptual structure, and their item selection is often driven by pragmatic convenience rather than conceptual mapping [61]. Value internalization is addressed partially in attitudinal subscales but is frequently conflated with knowledge items or treated interchangeably with awareness and cognition, producing blurred dimensional boundaries and reducing interpretability. Law-based situational practice is the attribute most closely tied to the occupational context of nursing, yet it is also the least adequately measured. Most existing tools focus on what nurses know or say about legal issues, rather than on whether they can identify legal risks, judge normative boundaries, choose procedurally appropriate responses, and act in a compliant manner in complex and time-pressured clinical situations. Because this attribute is absent from most current measures, the instruments cannot fully capture the construct they purport to assess, nor can they provide the dimensional structure needed for differential diagnosis of deficits or targeted educational intervention.

#### 3.6.3. Observable Empirical Referents by Defining Attribute

In accordance with the concept analysis framework, the following observable referents correspond to each defining attribute and provide the observable basis for future instrument development.

Empirical referents corresponding to normative understanding include nurses’ demonstrated comprehension of laws, regulations, institutional norms, procedural requirements, and responsibility boundaries in nursing practice, specifically, knowledge of nurses’ rights and obligations, patient-rights protection, informed consent procedures, privacy and confidentiality standards, nursing documentation requirements, boundaries of order execution authority, and procedures for managing disputes and adverse events [13,17,25,35,37].

Empirical referents of value internalization include whether nurses recognize legal norms as both guiding and constraining, actively consider legal and institutional requirements when making clinical decisions under uncertainty, show a sense of responsibility and motivation to comply with procedures, and view the protection of patient rights as a substantive professional obligation rather than a merely formal one [10,11,28,38,39,40].

Empirical referents corresponding to law-based situational practice include nurses’ performance across legal-practice intersections in clinical contexts: compliant identity verification, disclosure, and order execution; accurate and timely documentation; lawful privacy-protecting communication; appropriate identification and escalation of risk events; and normatively appropriate conduct in the early stages of potential disputes. These referents require scenario-based, situation-embedded, or behaviorally anchored assessment approaches rather than factual-recall formats [43,44].

### 3.7. Operational Definition of Legal Literacy in Clinical Nurses

Based on the results of the concept analysis, legal literacy in clinical nurses is defined as a multidimensional professional competency expressed in nursing practice, comprising: (1) the capacity to understand laws, regulations, institutional rules, and procedural requirements relevant to clinical nursing, and to use this understanding to identify legal issues and boundaries (legal knowledge reserve); (2) the stable internalization of legal demands concerning patient-rights protection, lawful practice, and professional responsibility as operative professional value orientations (clinical legal awareness); and (3) the capacity to identify legal issues, reason about normative boundaries, select procedurally appropriate responses, and implement compliant conduct in the concrete situations of clinical nursing practice (practice application ability). These three dimensions are conceptually distinct and empirically separable, yet mutually constitutive: together they constitute the integrated professional competency that is legal literacy in clinical nurses.

## 4. Discussion

This concept analysis was conducted to clarify the conceptual boundaries, defining attributes, antecedents, consequences, empirical referents, and operational definition of legal literacy in clinical nurses. The results represent a systematic conceptual structure that moves from cognitive foundation through professional value orientation to situational action capacity. The following discussion addresses the theoretical grounding and distinctiveness of the three defining attributes, the conceptual positioning of legal literacy within the broader literacy scholarship, the implications of the antecedent-consequence structure for nursing education and organizational management, the nature and extent of the measurement gap, and the limitations of the present analysis.

### 4.1. Theoretical Grounding and Distinctiveness of the Defining Attributes

The three defining attributes identified in this analysis—normative understanding, value internalization oriented toward rights and responsibilities, and law-based situational practice—should not be interpreted as a simple list of competencies. Rather, they form an internal developmental structure through which legal norms are first understood, then internalized as professional obligations, and finally translated into lawful action in clinical situations. This structure helps explain why legal literacy in clinical nurses cannot be reduced to legal knowledge alone. Although gaps in nurses’ legal knowledge have been documented across areas such as informed consent, privacy protection, documentation standards, duty-of-care boundaries, and adverse-event management [62,63], knowledge by itself does not necessarily lead to corresponding attitudinal orientation or legally appropriate practice behavior [64].

This three-attribute structure also clarifies the relationship between legal literacy and adjacent constructs. Legal knowledge provides the cognitive foundation; legal awareness reflects value-oriented endorsement of lawful practice; and legal cognition involves subjective understanding of the meaning and relevance of legal norms. Legal literacy, however, integrates these elements and extends them into accountable clinical action. The attribute of law-based situational practice is therefore particularly important, because it distinguishes clinical nurses’ legal literacy from general legal literacy or legal awareness. Evidence that scenario-based and malpractice-case educational approaches improve patient safety competency and critical thinking further supports the need to understand legal literacy as a situational and practice-oriented competency, rather than as factual legal knowledge alone [45].

The structure identified here also parallels broader literacy theory. Similar to Nutbeam’s distinction between functional, interactive, and critical health literacy [65], legal literacy in clinical nursing can be understood as progressing from foundational comprehension, to professional orientation, and finally to applied judgment and action. This comparison reinforces the need to measure and strengthen legal literacy as a multidimensional professional competency rather than through knowledge-based assessment alone.

### 4.2. Implications of the Antecedent-Consequence Structure for Nursing Education, Management, and Policy

The antecedent-consequence framework identified in this analysis has practical implications for nursing education and training, organizational risk management, quality governance, and policy development. It suggests that legal literacy in clinical nurses should not be addressed merely as an individual knowledge deficit, but as a multidimensional professional competency shaped by educational preparation, organizational conditions, and regulatory expectations.

At the level of nursing education, the three-attribute model indicates that legal education should adopt a differentiated and multidimensional design rather than a single-format knowledge-delivery approach. Normative understanding can be developed through structured instruction on laws, regulations, institutional rules, and procedural requirements. However, value internalization requires educational strategies that explicitly address professional identity, responsibility orientation, patient-rights protection, and moral-normative commitment. Such aims are more likely to be achieved through case-based ethical-legal discussion, reflective learning, and integration of legal obligations into professional value formation than through didactic teaching alone. Law-based situational practice, which is often insufficiently addressed in current educational approaches, requires scenario-based, situation-embedded, and behaviorally anchored learning methods. Yi and Kim [66] found in a randomized controlled trial that blended learning combining mock trials of medical malpractice cases with web-based education produced greater improvements in patient safety competency and critical thinking than web-based education alone. This finding supports the view that legal education should be evaluated not only by knowledge acquisition, but also by its ability to strengthen value orientation and legally appropriate action in realistic clinical situations.

The developmental nature of legal literacy also has implications for the timing and differentiation of education across nurses’ professional trajectories. Several studies have identified years of practice, specialty context, and prior exposure to disputes as important antecedents of nurses’ legal literacy or related legal competencies [52,53,54,55]. This suggests that legal education should not be confined to pre-registration training or delivered as a one-time intervention. Instead, it should be organized as a career-long, continuously updated professional development process. Specialty-specific legal education is particularly important for areas such as intensive care, emergency nursing, palliative care, pediatric nursing, and mental health nursing, where nurses encounter distinct legal obligations, high-risk decisions, and context-specific accountability requirements.

At the organizational level, the antecedent analysis shows that the translation of legal knowledge and value internalization into law-based situational practice depends heavily on institutional risk governance. Legal literacy cannot be sustained by individual education alone; it requires organizational systems that provide accessible and updated legal standards, clear protocols for high-risk areas such as informed consent, privacy protection, adverse-event management, documentation, and dispute handling, as well as timely legal and managerial consultation. Organizational cultures characterized by punitive blame orientation may discourage the very behaviors central to law-based situational practice, including incident reporting, accurate documentation of adverse events, and lawful disclosure to patients and families [67]. Therefore, strengthening nurses’ legal literacy requires organizational preconditions such as non-punitive reporting systems, regular case-based review of legal risk events, and governance structures that support transparency, accountability, and procedural compliance.

At the level of policy and regulatory development, the operational definition produced by this concept analysis provides a conceptual basis for incorporating legal literacy as a formally specified dimension within nursing competency standards, pre-registration curricula, and continuing professional development frameworks. The operational definition formulated in Section 3.7: comprising legal knowledge reserve, clinical legal awareness, and practice application ability, provides the dimensional structure for incorporating legal literacy into competency standards in a way that specifies not only what nurses should know but what they should be oriented toward and what they should be able to do in specific clinical situations.

### 4.3. Empirical Referents, the Measurement Gap, and Implications for Instrument Development

The analysis of empirical referents in Section 3.6 revealed a systematic and persistent measurement gap that mirrors a well-documented problem in the broader literacy measurement literature: existing instruments are predominantly concentrated at the level of normative understanding, inconsistently and ambiguously operationalize value internalization, and largely fail to capture law-based situational practice as a distinct and measurable dimension.

A fundamental concern with the dominant measurement paradigm in this field is that it has relied on a knowledge-attitude-practice (KAP) framing that does not correspond to the three-attribute conceptual structure identified in this analysis. Instruments operationalizing “legal awareness,” “legal cognition,” or “legal literacy” using factual knowledge items assess normative understanding but treat it as equivalent to the fuller construct; instruments adding attitudinal subscales are closer to capturing value internalization but typically fail to distinguish it from awareness or perception of risk; and neither type of instrument adequately operationalizes the situational judgment and behavioral enactment dimensions of law-based situational practice. This pattern is structurally parallel to what has been extensively documented in the health literacy field: health literacy is broadly defined as encompassing information-seeking, decision-making, problem-solving, and critical thinking, but measured with instruments that focus predominantly on basic reading and comprehension tasks, producing a systematic mismatch between conceptualization and operationalization. In the nursing legal literacy field, the same mismatch operates: the concept is implicitly defined broadly in the literature, but measured narrowly through factual recall instruments that capture only its most basic cognitive dimension.

The consequences of this measurement gap are multidirectional. First, the construct validity of existing legal literacy measures is compromised, because instruments that assess only normative understanding cannot distinguish between nurses who possess legal knowledge but apply it inconsistently in practice from those who demonstrate stable, situation-appropriate legal conduct. Second, the comparability of findings across studies is systematically limited, because the heterogeneity of construct operationalization means that studies labeled as assessing “legal literacy,” “legal awareness,” and “legal knowledge” may be measuring structurally different things. Third, and most consequentially for practice, the educational and management interventions derived from these measures are likely to be inadequately targeted, because the deficit dimension most relevant to patient safety and legal risk prevention (law-based situational practice) is precisely the one that existing tools systematically fail to measure.

The operational definition and defining attributes established in this concept analysis directly address these limitations by providing a theoretically grounded dimensional structure for instrument development. A valid measurement instrument for legal literacy in clinical nurses must operationalize all three defining attributes through distinct item pools and, ideally, distinct assessment formats appropriate to each dimension. Normative understanding can be assessed through knowledge-based items covering the legally regulated areas of clinical nursing practice. Value internalization requires orientation-based items that assess nurses’ endorsement of patient-rights protection as an operative professional obligation and their proactive tendency to reference legal and institutional requirements in clinical decision-making under uncertainty. Law-based situational practice requires scenario-based, behaviorally anchored items that present realistic nursing situations involving legal risk and assess nurses’ capacity to identify the legal issue, reason about appropriate responses, and select the procedurally correct course of action. Such an instrument, grounded in the conceptual structure provided by this analysis, would constitute the next essential step in developing the evidence base for nursing legal education and management. The instrument development process should include item generation informed by the defining attributes and empirical referents identified here, expert content validity assessment by panels including nursing clinicians, nursing educators, and healthcare legal experts, cognitive interviewing with clinical nurses, and psychometric validation of the three-factor structure in adequately powered clinical samples.

### 4.4. Limitations

Several limitations of this concept analysis should be acknowledged. First, although the literature search covered six major databases and was supplemented by searches in legal and social science sources, a prospective protocol was not registered in a registry such as PROSPERO, and the possibility that relevant sources were not captured cannot be fully excluded. Second, the defining attributes, cases, antecedents, consequences, and empirical referents identified in this analysis represent a conceptual structure derived from the current state of the published literature; because legal literacy in clinical nurses is a dynamic concept shaped by evolving legislation, shifting healthcare delivery models, and changing public expectations of nursing accountability, its constitutive features may require periodic re-examination as the regulatory and professional context develops. Third, while the regulatory and institutional context of Chinese healthcare is present in a substantial portion of the included literature, the operational definition and defining attributes were derived from a synthesis of international sources, and the extent to which the operational definition applies uniformly across healthcare systems with different legal traditions and nursing scope-of-practice frameworks requires further cross-cultural validation. Fourth, Walker and Avant’s method is primarily an analytical rather than an empirical approach; the cases constructed in this analysis are illustrative scenarios designed to test the defining attributes, rather than observations drawn from clinical practice, and should not be interpreted as epidemiologically representative of the distribution of legal literacy competence in clinical nurse populations.

## 5. Conclusions

Using Walker and Avant’s method of concept analysis, this study systematically clarified the connotation of legal literacy in clinical nurses. Based on an analysis of 56 papers, it clarified the conceptual boundaries of legal literacy in clinical nurses, identified its defining attributes, and articulated an internal structure that moves from cognitive foundation (normative understanding), to value orientation (value internalization oriented toward rights and responsibilities), and then to situational practice (law-based situational practice). The study also analyzed the antecedents, consequences, and empirical referents of legal literacy in clinical nurses at multiple levels, and on that basis formulated an operational definition that distinguishes three operationalizable dimensions: legal knowledge reserve, clinical legal awareness, and practice application ability.

The findings make two principal contributions to nursing science. Conceptually, they provide a theoretically grounded and terminologically precise account of legal literacy in clinical nurses that resolves the conflation of related but distinct constructs pervading the existing literature. Methodologically, the systematic identification of empirical referents and the critical evaluation of existing instruments reveal a persistent measurement gap—particularly in the law-based situational practice dimension—that directly motivates the development of a multidimensional, three-factor assessment scale. Such an instrument would enable valid measurement, differential diagnosis of competence deficits across the three dimensions, rigorous evaluation of educational interventions, and cross-institutional benchmarking. These results provide a theoretical basis for understanding legal literacy in clinical nurses and a conceptual and structural foundation for subsequent measurement development, targeted legal education, and evidence-based nursing management.

## Data Availability

The original contributions presented in this study are included in the article and Supplementary Materials. Further inquiries can be directed to the corresponding author.

## Supplementary Materials

The following supporting information can be downloaded at: https://www.mdpi.com/article/10.3390/nursrep16060200/s1, Table S1: Database-specific search strategies for the primary search; Table S2: Summary of included sources and their relevance to the concept analysis [5,9,10,12,14,15,16,17,24,38,39,53,54,60,64,68,69,70,71,72,73,74,75,76,77,78,79,80,81,82,83,84,85,86,87,88,89,90,91,92,93,94,95,96,97,98,99,100,101,102,103,104,105,106,107,108].

## Public Involvement Statement

No public involvement in any aspect of this research.

## Guidelines and Standards Statement

This manuscript was drafted in accordance with Walker and Avant’s eight-step method for concept analysis [21]. The literature identification, screening, and reporting process was informed by the Preferred Reporting Items for Systematic Reviews and Meta-Analyses (PRISMA) 2020 statement [22].
