# Supplementary material for: Legal Literacy in Clinical Nursing Practice: A Walker and Avant Concept Analysis
Source: Nurs Rep. 2026 Jun 12;16(6):200. doi: 10.3390/nursrep16060200 (PMC13304500; doi:10.3390/nursrep16060200)
Supplement: Supplementary file 1 [file nursrep-16-00200-s001.zip › nursrep-4295818-supplementary.pdf]

## Supplementary Table S1. Database-specific search strategies for the primary search

The complete database-specific search strategies for the primary search are provided below.

| Database | Search field | Search syntax | Date searched | Records identified |
|---|---|---|---|---|
| Wanfang | Title/Abstract/Keywords | ("legal literacy" OR "legal knowledge" OR "legal awareness" OR "legal cognition" OR "legal responsibility" OR "medical law" OR "health law" OR "nursing law" OR "legal obligation" OR "professional responsibility")<br>AND<br>("nurse" OR "nursing" OR "clinical nurse" OR "medical staff" OR "healthcare personnel" OR "clinical practice") | December 2024 | 276 |
| VIP | Title/Abstract/Keywords | ("legal literacy" OR "legal knowledge" OR "legal awareness" OR "legal cognition" OR "legal responsibility" OR "medical law" OR "health law" OR "nursing law" OR "legal obligation" OR "professional responsibility")<br>AND<br>("nurse" OR "nursing" OR "clinical nurse" OR "medical staff" OR "healthcare personnel" OR "clinical practice") | December 2024 | 401 |
| CNKI | Title/Abstract/Keywords | ("legal literacy" OR "legal knowledge" OR "legal awareness" OR "legal cognition" OR "legal responsibility" OR "medical law" OR "health law" OR "nursing law" OR "legal obligation" OR "professional responsibility")<br>AND<br>("nurse" OR "nursing" OR "clinical nurse" OR "medical staff" OR "healthcare personnel" OR "clinical practice") | December 2024 | 243 |
| CINAHL | Title/Abstract/Subject terms | ("legal literacy" OR "legal knowledge" OR "legal awareness" OR "legal cognition" OR "legal liability" OR "medical law" OR "health law" OR "nursing law" OR "legal responsibility" OR "professional responsibility")<br>AND<br>("nurse*" OR "nursing" OR "clinical nurse*" OR "healthcare professional*" OR "clinical practice") | December 2024 | 285 |
| Cochrane Library | Title/Abstract/Keyword | ("legal literacy" OR "legal knowledge" OR "legal awareness" OR "legal cognition" OR "legal liability" OR "medical law" OR "health law" OR "nursing law" OR "legal responsibility" OR "professional responsibility")<br>("nurse*" OR "nursing" OR "clinical nurse*" OR "healthcare professional*" OR "clinical practice") | December 2024 | 7 |
| Embase | Title/Abstract/Keywords | ('legal literacy':ti,ab,kw OR 'legal knowledge':ti,ab,kw OR 'legal awareness':ti,ab,kw OR 'legal cognition':ti,ab,kw OR 'legal liability':ti,ab,kw OR 'medical law':ti,ab,kw OR 'health law':ti,ab,kw OR 'nursing law':ti,ab,kw OR 'legal responsibility':ti,ab,kw OR 'professional responsibility':ti,ab,kw)<br>AND<br>(nurs*:ti,ab,kw OR 'nursing':ti,ab,kw OR 'clinical nurse':ti,ab,kw OR 'healthcare professional':ti,ab,kw OR 'clinical practice':ti,ab,kw) | December 2024 | 234 |
| PubMed | Title/Abstract | ("legal literacy"[Title/Abstract] OR "legal knowledge"[Title/Abstract] OR "legal awareness"[Title/Abstract] OR "legal cognition"[Title/Abstract] OR "legal liability"[Title/Abstract] OR "medical law"[Title/Abstract] OR "health law"[Title/Abstract] OR "nursing law"[Title/Abstract] OR "legal responsibility"[Title/Abstract] OR "professional responsibility"[Title/Abstract])<br>AND<br>("nurse*"[Title/Abstract] OR "nursing"[Title/Abstract] OR "clinical nurse*"[Title/Abstract] OR "healthcare professional*"[Title/Abstract] OR "clinical practice"[Title/Abstract]) | December 2024 | 159 |
| Web of Science | Topic | TS=("legal literacy" OR "legal knowledge" OR "legal awareness" OR "legal cognition" OR "legal liability" OR "medical law" OR "health law" OR "nursing law" OR "legal responsibility" OR "professional responsibility")<br>AND<br>TS=("nurse*" OR "nursing" OR "clinical nurse*" OR "healthcare professional*" OR "clinical practice") | December 2024 | 163 |

**Note.** Search syntax was adapted according to the indexing rules and available search fields of each database. The supplementary conceptual search using "legal consciousness" was conducted to clarify the boundary between legal awareness and legal consciousness and did not yield additional sources eligible for inclusion in the primary concept analysis.

The search terms originally used in Chinese databases have been translated into English for presentation in the supplementary materials.

## Supplementary Table S2. Summary of included sources and their relevance to the concept analysis

This table provides a detailed summary of the included sources and their relevance to the identification of defining attributes, antecedents, consequences, empirical referents, and conceptual boundaries.

**Note.** Chinese-language source titles have been translated into English. English-language source titles are retained as originally published.

| No. | Source title | Source information | Main focus | Relevance to the present concept analysis |
|---|---|---|---|---|
| 1 | Analysis and prevention of legal risks in obstetric nursing under the drafting background of the Nurses Law | Gao, L, 2016; theoretical discussion; Mainland China | Analyzed causes of legal risks in obstetric nursing and proposed prevention strategies involving privacy, informed consent, nursing documentation, nursing skills, and service attitude. | Antecedent: insufficient nursing legal education. Empirical referents: privacy protection, informed consent, nursing records, and risk-prevention behaviors. Consequences: nursing disputes and impaired nurse–patient relationships. |
| 2 | Current status and influencing factors of nursing-related legal risk cognition among intern nurses in Changde | Lin, C.J. 2016; thesis; Mainland China | Surveyed intern nurses' cognition of nursing-related legal risks, analyzed influencing factors, and proposed training recommendations. | Antecedents: individual and training-related factors. Conceptual boundary: helps distinguish legal risk cognition from legal literacy. |
| 3 | Problems and countermeasures regarding evidence production by emergency nurses in nurse–patient disputes | Dong, J.Y., 2009; theoretical discussion; Mainland China | Analyzed problems faced by emergency nurses in providing evidence during nurse–patient disputes and proposed countermeasures, focusing on nursing records, evidence awareness, and compliant practice. | Empirical referents: completeness of nursing records, evidence preservation, and standardized practice. Consequence: insufficient evidence may increase liability risk. Defining attribute: supports law-based situational practice. |
| 4 | Legal responsibilities of nursing personnel and risk-prevention measures | Ge, X.C.， 2004; theoretical discussion; Mainland China | Discussed possible criminal, civil, and administrative responsibilities of nursing personnel and proposed risk-prevention measures. | Conceptual boundary: distinguishes legal responsibility, legal awareness, and legal literacy. Defining attributes: supports normative understanding and value internalization. Consequence: legal responsibility and risk exposure. |
| 5 | Knowledge, attitudes, and behaviors of nursing personnel regarding nurse–patient disputes | Yan, X., 2011; cross-sectional study; Mainland China | Investigated nurses' knowledge of nurse–patient disputes, attitudes toward disputes, and defensive nursing behaviors. | Defining attributes: supports normative understanding, value internalization, and law-based situational practice. Empirical referents: dispute-related knowledge, dispute attitudes, and defensive nursing behaviors. Supports the knowledge–orientation–practice structure of the operational definition. |
| 6 | Current status of nurses' legal awareness and countermeasures | Basang; Deng, Z., 2005; theoretical discussion; Mainland China | Discussed nurses' legal awareness in relation to nursing errors, increased patient rights awareness, and insufficient legal education, and proposed improvement measures. | Conceptual boundary: supports the distinction between legal awareness and legal literacy. Antecedents: insufficient legal courses in nursing education and increased patient rights awareness. Defining attribute: supports value internalization. |
| 7 | Status of legal knowledge mastery among nursing personnel and its influencing factors. | Tian, S.N., 2013; cross-sectional study; Mainland China | Investigated nurses' mastery of legal knowledge and analyzed influencing factors such as professional title, position, years of work, and education level. | Conceptual boundary: supports legal knowledge as a foundation of legal literacy but not equivalent to the full construct. Antecedents: education, professional title, position, and years of work. Empirical referent: level of legal knowledge. |
| 8 | Knowledge level and training regarding occupation-related laws and regulations among nurses | Liu, Y. , 2022; cross-sectional study; Mainland China | Conducted a national survey of nurses' knowledge of laws and regulations and training status, analyzing hospital level, years of work, education, professional title, and training factors. | Antecedents: school education, induction training, institutional support, and professional experience. Defining attribute: supports normative understanding. |
| 9 | Characteristics and main content of the Nurses Regulations III: Responsibilities and legal liabilities of governments and healthcare institutions. | Liu, X., 2008; regulatory interpretation; Mainland China | Interpreted provisions of the Nurses Regulations regarding government, healthcare institution, and nursing practice management responsibilities, emphasizing training, practice management, and legal responsibility. | Antecedents: legal environment, institutional norms, and practice management requirements. Conceptual boundary: clarifies nurses' rights, obligations, and responsibility boundaries. Operational definition: provides institutional normative basis. |
| 10 | Causes of nursing practice disputes: an analysis of court cases | Chou, H.J., 2010; case analysis; Taiwan, China | Analyzed causes of nursing practice disputes based on court cases, including care negligence, negligent execution of medical assistance, actions beyond nursing scope, and administrative negligence. | Empirical referents: care negligence, improper order execution, boundary-crossing behavior, and administrative negligence. Consequences: nursing disputes and litigation. Defining attribute: supports law-based situational practice. |
| 11 | Legal risk management in nursing practice: Focusing on duty of care | Yang, W.P, 2021; topical review; Taiwan, China | Discussed legal risk management in nursing practice centered on duty of care, including care duty, duty in medical assistance, management and monitoring duties, and related cases. | Defining attribute: supports law-based situational practice. Empirical referents: performance of duty of care, order execution, risk management, and monitoring. Operational definition: supports legal identification, judgment, and compliant response in clinical situations. |
| 12 | Current status of nursing-related legal education and legal literacy among clinical nurses | Wang, S.Y., 2023; cross-sectional study; Mainland China | Investigated clinical nurses' legal education, legal knowledge, and legal literacy, and analyzed influencing factors across eight common nursing legal topics, including informed consent, privacy, and emergency care. | Defining attributes: supports normative understanding and law-based situational practice. Empirical referents: performance on topics such as informed consent, privacy, and emergency care. Conceptual boundary: indicates that legal knowledge and legal literacy are not fully equivalent. |
| 13 | On the legal responsibilities of practicing nurses | Niu, Y.P., 2005; theoretical discussion; Mainland China | Discussed legal responsibilities of practicing nurses, including practice qualification, patient life safety, order execution, nursing records, medication management, and privacy protection. | Conceptual boundary: distinguishes legal responsibility from legal literacy. Defining attributes: supports normative understanding and law-based situational practice. Consequences: infringement, liability, and impairment of patient rights. |
| 14 | Effect of the 'three constants and two stricts' method in legal knowledge training for nurses in joint-stock hospitals. 2012 | Zhang, W.L., 2012; quasi-experimental study; Mainland China | Evaluated systematic legal knowledge training for nurses using the "three constants and two stricts" method and compared legal knowledge before and after training across education levels. | Antecedent: in-service training and institutional legal education support are important conditions for legal literacy. Empirical referent: legal knowledge test results. Consequence: systematic training can improve nurses' legal knowledge. |
| 15 | Survey of legal awareness among clinical nurses in tertiary Grade A hospitals | Qu, J.B., 2009; cross-sectional study; Mainland China | Surveyed legal awareness among clinical nurses in tertiary Grade A hospitals and analyzed years of nursing, professional title, education, and sources of legal knowledge. | Conceptual boundary: helps distinguish legal awareness from legal literacy. Defining attribute: mainly supports value internalization. Antecedents: school education, hospital education, and self-learning. |
| 16 | Analysis of Medical Legal Knowledge Awareness Rate and Communication Ability Among Nurses in a Tertiary Hospital in Shenyang | Chang, Y.X., 2018; thesis; Mainland China | Investigated nurses' awareness of medical legal knowledge, humanistic ethics, and communication ability in a tertiary hospital and analyzed influencing factors. | Conceptual boundary: distinguishes legal knowledge, communication ability, humanistic ethics, and legal literacy. Antecedents: school education and institutional training. Empirical referents: responses to legal knowledge, ethics, and communication items. |

| No. | Source title | Source information | Main focus | Relevance to the present concept analysis |
|---|---|---|---|---|
| 17 | Legal risks and recommendations regarding multi-site practice for nurses in China. | Liu, Q.X., 2020; policy analysis; Mainland China | Analyzed legal risks in multi-site nursing practice, including legality, responsibility allocation, nursing quality, and regulation, and proposed institutional recommendations. | Antecedents: legal environment, institutional norms, and practice management requirements. Defining attributes: supports normative understanding and law-based situational practice. Consequences: impaired nursing quality, unclear responsibility, and increased legal risk. |
| 18 | Survey of new nurses' cognition and needs regarding legal knowledge | Sheng, L.J., 2015; cross-sectional study; Mainland China | Surveyed new nurses' cognition of health laws and regulations, sources of legal knowledge, and learning needs. | Antecedents: school legal education, induction training, and case-based teaching needs. Empirical referents: legal knowledge cognition scores and learning needs. Conceptual boundary: supports legal knowledge as a foundational but incomplete dimension of legal literacy. |
| 19 | Survey of legal literacy among nurses in tertiary Grade A hospitals in Xuzhou. | Wang, X.Y., 2018; cross-sectional study; Mainland China | Investigated nurses' legal literacy and analyzed relationships among legal knowledge, legal awareness, legal application, and demographic variables. | Defining attributes: directly supports normative understanding, value internalization, and law-based situational practice. Empirical referents: legal knowledge, legal awareness, and legal application. Antecedents: professional title, education, age, and working time. |
| 20 | Current status and influencing factors of legal risk cognition among community nurses in Changsha-Zhuzhou-Xiangtan | Tan, F.L., 2009; thesis; Mainland China | Investigated community nurses' cognition and attitudes toward health laws and regulations and analyzed legal risks in community nursing behaviors that may trigger nurse–patient disputes. | Antecedents: age, education, professional title, and transfer training. Empirical referents: nursing records, home care, health education, and order execution as high-risk links. Conceptual boundary: legal risk cognition is related to but not identical with legal literacy. |
| 21 | Importance of rule-of-law education in medical talent training under the New Medical Science strategy | Zhou, C.C., 2022; policy analysis; Mainland China | Discussed the need to integrate rule-of-law education into medical education under the New Medical Science strategy, focusing on expanding medical risks, increased patient rights awareness, intelligent technologies, and the neglect of humanities in medical education. | Antecedent: changes in the rule-of-law environment. Operational definition: supports legal literacy as a comprehensive capability needed by healthcare professionals when facing medical risks, rights and obligations, and normative problems. Conceptual boundary: clarifies relationships among rule-of-law education, legal literacy, and professional competence. |
| 22 | A Placebo for the Pain: A Medico-legal Case Analysis | Ben A. Rich, 2003; medico-legal case analysis; United States | Analyzed the deceptive use of placebo in pain management through a case of adolescent migraine from clinical, ethical, and legal perspectives, including informed consent, professional responsibility, and legal consequences of nursing behavior. | Defining attributes: supports normative understanding and law-based situational practice. Empirical referents: informed consent, pain management, order execution, and compliance judgment. Consequences: professional complaints, licensure review, and liability disputes. |
| 23 | A story of scrutiny and fear: Australian midwives' experiences of an external review of obstetric services | Laraine Hood, 2010; qualitative study; Australia | Explored midwives' experiences after external scrutiny and legal processes, identifying themes of scrutiny and fear and describing effects on practice and personal life. | Antecedents: litigation environment, external review, and exposure to legal procedures. Consequences: defensive decision-making, altered practice, and damaged professional relationships. |
| 24 | Angels of Mercy? The Legal and Professional Implications of Withdrawal of Life-Sustaining Treatment by Nurses in England and Wales | Giles Birchley, 2012; topical review; United Kingdom (England and Wales) | Examined nurses' legal and professional responsibilities in withdrawing life-sustaining treatment, including differences between physicians and nurses in common law protection, professional responsibility, and regulatory risk. | Conceptual boundary: distinguishes legality, professional responsibility, and professional autonomy. Defining attributes: supports normative understanding and role responsibility. Operational definition: indicates that legal literacy involves role-specific judgment rather than legal knowledge alone. |
| 25 | Comparison of educational needs and priorities for work-related laws between nurses in healthcare institutions and community settings | Jeonghyun Kim, 2025; cross-sectional study; South Korea | Compared educational needs and priorities for work-related laws among nurses in healthcare institutions and community settings, highlighting practical difficulties caused by insufficient legal knowledge and differences across work settings. | Antecedents: work-setting differences, insufficient legal education, and unclear legal responsibility. Defining attributes: supports normative understanding and practice application. Empirical referents: role ambiguity, unclear practice scope, and difficulty responding to legal issues. |
| 26 | Current Legal Changes: Innovative Legal Seminar for Nursing Students | Irene E. Pappas, 2007; topical review; United States | Described the integration of legal seminars into undergraduate nursing curricula, emphasizing nurse-attorney involvement to improve understanding of health law, negligence, documentation, and end-of-life issues. | Antecedents: insufficient updates in legal education and limited legal training in nursing curricula. Defining attribute: supports normative understanding. Empirical referent: ability to identify legally significant nursing situations and respond appropriately. |
| 27 | Discussing the Limits of Confidentiality: The Impact of Criminalizing HIV Non-disclosure on Public Health Nurses' Counseling Practices | Chris Sanders, 2014; qualitative study; Canada | Analyzed how public health nurses manage boundaries of confidentiality, counseling practice, and legal risk in the context of criminalized HIV non-disclosure. | Defining attributes: supports law-based situational practice and value internalization. Empirical referents: disclosure of confidentiality limits, risk communication, and management of confidentiality exceptions. Antecedent: criminalization and judicial intervention in nursing counseling practice. |
| 28 | Effect of mobile-based education of professional laws and related legal punishments on knowledge, attitude, and performance of operating room nurses | Fardin Amiri, 2026; quasi-experimental study; Iran | Evaluated mobile-based legal education for operating room nurses using a one-group pretest–posttest design; knowledge, attitudes, and performance improved after intervention. | Antecedent: continuing legal education and training. Defining attributes: supports normative understanding, value orientation, and practical performance. Empirical referents: knowledge, attitude, and performance scores. |
| 29 | Emergency department and intensive care unit health professionals' knowledge and attitudes regarding end-of-life law | Jayne Hewitt, 2023; scoping review; Australia | Reviewed emergency and ICU health professionals' knowledge, attitudes, and use of end-of-life law, identifying substantial legal knowledge gaps and the tendency for legal factors to be secondary to clinical judgment. | Defining attributes: supports normative understanding and law-based situational practice. Antecedent: legal complexity. Consequence: concern about legal risk may contribute to continuation of potentially non-beneficial end-of-life treatment. |
| 30 | Improving Baccalaureate Nursing Students' Understanding of Fundamental Legal Issues Through Interdisciplinary Collaboration | Chad Priest, 2007; topical review; United States | Described interdisciplinary collaboration among nursing faculty, nurse-attorneys, and undergraduate students to improve understanding of patient rights, informed consent, privacy, and patient safety. | Antecedent: insufficient legal training in undergraduate education. Conceptual boundary: indicates links among patient advocacy, patient safety, and legal knowledge, while distinguishing them from full legal literacy. |

| No. | Source title | Source information | Main focus | Relevance to the present concept analysis |
|---|---|---|---|---|
| 31 | Interprofessional collaboration and health policy: results from a Quebec mixed-methods legal study | Marie-Andrée Girard, 2022; mixed-methods legal study; Canada (Quebec) | Combined normative text analysis and survey research to examine nurses' and physicians' knowledge of the legal framework for interprofessional collaboration, sources of legal knowledge, and perceptions of responsibility. | Antecedents: policy environment and organizational norms. Defining attribute: supports normative understanding. Consequences: misunderstanding law and responsibility may impede interprofessional collaboration and increase responsibility concerns. |
| 32 | Ivory Tower or Trench? A Q Study of Legal Knowledge and Skills for Graduate Nurses | Terri L. Frock, 1994; doctoral dissertation; United States | Used Q methodology to compare views of nursing faculty and employers on legal knowledge and skills required of graduate nurses, revealing an education–practice gap. | Conceptual boundary: positions legal knowledge and skills as outcomes of nursing professional socialization. Operational definition: indirectly supports the relationship among knowledge, skills, and practice translation in legal literacy. |
| 33 | Medication safety challenges in primary care: Nurses' perspective | Hanan Khalil, 2018; qualitative study; Australia | Used semi-structured interviews to analyze nurses' perceptions of medication safety in community settings, identifying unclear roles, underreporting of errors, inadequate documentation, and insufficient training. | Antecedents: organizational culture, unclear role boundaries, and inadequate educational support. Empirical referents: error reporting, documentation, and medication safety responses. Consequences: patient safety harm and liability risk. |
| 34 | Knowledge and Attitude of Emergency Nurses Regarding Work-Related Legal Issues | Hardi Abdulqadir Hasan, 2025; cross-sectional study; Iraq | Assessed emergency nurses' legal knowledge, legal attitudes, and involvement in legal issues, and analyzed their relationships. | Defining attributes: supports normative understanding and value internalization. Empirical referents: legal knowledge score, attitude score, and legal issue involvement. Conceptual boundary: shows legal knowledge, attitudes, and practice difficulties as related but distinguishable dimensions. |
| 35 | Knowledge and Perceptions of Health Workers' Training on Ethics, Confidentiality and Medico-Legal Issues | Bernard Asamoah Barnie, 2015; cross-sectional study; Ghana | Investigated health workers' knowledge and perceptions of training in ethics, confidentiality, and medico-legal issues, focusing on shortcomings in formal and in-service education. | Antecedents: insufficient legal and ethical training and limited curricula. Defining attributes: supports normative understanding and value orientation. Empirical referents: responses on confidentiality, medico-legal concepts, and training needs. |
| 36 | Knowledge, Attitude And Practice (KAP) Study On Medicolegal Knowledge Of Healthcare Professionals In Cases Of Poisoning | Radhika Hande, 2025; cross-sectional study; India | Investigated healthcare professionals' knowledge, attitudes, and practice in medico-legal management of poisoning cases, focusing on reporting, sample preservation, chain of custody, and documentation quality. | Defining attributes: supports normative understanding and law-based situational practice. Empirical referents: reporting, sample sealing, chain-of-custody maintenance, and documentation. Antecedents: inadequate continuing medical education and hospital protocols. Consequences: impaired judicial procedures and liability risk. |
| 37 | The Nurse and the Law: A Primer | Paula DiMeo Grant, 2011; book; United States | Introduced the relationship between nursing and law, nurse practice acts, licensure, ethical concepts, legal obligations, and regulatory frameworks. | Conceptual boundary: clarifies law, ethics, obligation, and accountability. Defining attributes: supports normative understanding and responsibility awareness. Operational definition: contributes to the legal foundation of nursing practice. |
| 38 | Legal competence of emergency nurses / Competência legal do enfermeiro na urgência | Luiz Alves Morais Filho, 2016; qualitative study; Brazil | Reviewed nursing council resolutions, opinions, and decisions to clarify which emergency and urgent care procedures are within nurses' legal competence, which are not, and which are permitted only in life-threatening situations. | Defining attributes: supports normative understanding and law-based situational practice. Empirical referent: whether specific clinical behaviors are legally compliant. |
| 39 | Legal Awareness and Responsibilities of Nursing Staff in Administration of Patient Care in a Trust Hospital | Hemant Kumar, 2013; cross-sectional pilot study; India | Investigated nurses' legal awareness, basic legal knowledge, and legal responsibilities in basic nursing procedures, comparing age, experience, and education groups. | Conceptual boundary: supports distinguishing legal awareness from legal knowledge. Defining attribute: mainly supports normative understanding. Empirical referents: patient rights, legal duties, and cognition scores related to nursing procedures. Antecedent: lack of structured legal training. |
| 40 | Legal precedents and nursing: Implications of four key court findings for the nursing profession | Jonathan Bayuo, 2025; case law analysis; United States | Analyzed four recent court findings from legal databases and discussed implications for nursing jurisprudence, responsibility, transparency, patient rights, and regulatory policy. | Conceptual boundary: clarifies nursing jurisprudence, accountability, and patient rights. Antecedent: changes in case law may reshape nursing practice and legal expectations. |
| 41 | Medical futility in children's nursing: making end-of-life decisions | Irene O'Brien, 2010; topical review; Ireland | Discussed legal and ethical relationships among medical futility, parental autonomy, and the child's best interests in pediatric end-of-life care, with reference to case law. | Defining attributes: supports value internalization and law-based situational practice. Empirical referents: end-of-life communication and legal–ethical judgment in withholding treatment contexts. |
| 42 | End-of-life care in UK critical care units – a literature review | Jane Morgan, 2008; literature review; United Kingdom | Reviewed legal, ethical, policy, and organizational support issues in end-of-life care in UK critical care units, emphasizing nursing roles after withholding or withdrawing treatment decisions. | Antecedents: policy, resource allocation, organizational support, and insufficient education. Defining attribute: supports law-based situational practice. Consequences: effects on end-of-life care quality, family acceptance, and patient dignity. |
| 43 | Online modules to improve health professionals' end-of-life law knowledge and confidence | Rachel Feeney, 2023; pre-post survey study; Australia | Evaluated online training modules designed to improve health professionals' knowledge of end-of-life law and confidence in legal application. | Antecedent: insufficient specialized legal training. Defining attribute: supports normative understanding. |
| 44 | Organ and Tissue Donation Knowledge Among Intensive Care Unit Nurses | A. P. Shabanzadeh, 2009; cross-sectional study; Iran | Assessed ICU nurses' knowledge and attitudes regarding brain death, organ donation law, judicial issues, economic perceptions, and organ donation. | Antecedent: insufficient education. Empirical referents: knowledge of brain death, organ donation law, and legal-judicial aspects. |
| 45 | Medical Ethics, Law and Communication at a Glance | Patrick Davey, 2017; book; United Kingdom | Introduced core concepts in medical ethics, medical law, and communication, including confidentiality, consent, end-of-life care, and resource allocation. | Conceptual boundary: distinguishes ethics, law, and communication. Defining attributes: supports value internalization and normative understanding. Operational definition: informs the relationship between legal literacy and adjacent professional competencies. |
| 46 | Perception of legal liability by registered nurses in Korea | Ki-Kyong Kim, 2007; cross-sectional study; South Korea | Investigated registered nurses' perceptions of legal liability, attitudes toward physicians' supervisory duties, and legal awareness, and analyzed relationships among these constructs. | Conceptual boundary: distinguishes legal awareness, liability, and physicians' duty to supervise nurses. Defining attributes: supports normative understanding and value orientation. |

| No. | Source title | Source information | Main focus | Relevance to the present concept analysis |
|---|---|---|---|---|
| 47 | Perceptions and practices of medical ethics and laws among health professionals in Abu Dhabi | Emad Abdel Rahim Dahiyat, 2025; cross-sectional study; United Arab Emirates | Investigated physicians' and nurses' perceptions and practices of medical ethics and law, focusing on patient wishes, informed consent, confidentiality, conflicts of interest, and continuing education. | Conceptual boundary: distinguishes law from ethics. Defining attributes: supports normative understanding, value internalization, and practical implementation. Empirical referents: informed consent implementation and confidentiality practice. |
| 48 | Rights and duties policy implementation in Chile: health-care professionals' perceptions | Constanza R. Barrera, 2015; qualitative descriptive study; Chile | Used semi-structured interviews to analyze healthcare professionals' perceptions of implementation of Chile's Patient Rights and Duties Law, barriers, and improvement suggestions. | Antecedents: insufficient institutional dissemination, scarce resources, inadequate training, and lack of organizational support. Empirical referents: awareness, implementation, and feedback regarding patient rights and duties law. Operational definition: highlights the institutional context of legal literacy. |
| 49 | The Necessity of Legal Awareness of the Nurses in Health System | Fatemeh Ghofrani-Kelishami, 2020; qualitative study; Iran | Used interviews with judges, forensic experts, nurse-attorneys, nurses, and forensic midwives to identify subthemes of nurses' legal awareness, including informed consent, nursing errors, nurses' rights, patients' rights, and insufficient documentation. | Antecedents: lack of legal knowledge and insufficient curriculum content. Defining attributes: supports normative understanding and rights-and-responsibilities awareness. Empirical referents: handling informed consent, nursing errors, patient rights protection, and nursing documentation quality. Consequences: legal disputes and professional risk. |
| 50 | The Nurse's Role in the Informed Consent Process | Pamela A. Rosse, 1999; narrative review; United States | Reviewed the development and elements of informed consent and nurses' roles and responsibilities in research and clinical contexts, emphasizing education, clarification, patient advocacy, and ongoing assessment. | Conceptual boundary: clarifies informed consent in relation to patient autonomy, beneficence, and patient advocacy. Defining attributes: supports normative understanding, value internalization, and law-based situational practice. |
| 51 | Clinical nurses' legal roles, challenges, and responses to enabling legislation for nurses: A qualitative study | Xiaolong Wang, 2024; qualitative study; Mainland China | Used semi-structured interviews to analyze clinical nurses' legal cognition, legal training needs, rights-protection demands, and expectations for Nurses Law legislation, identifying themes of nursing environment, legal training needs, and attitudes toward legislative empowerment. | Antecedents: insufficient legal training, limited social support, and unfavorable professional environment. Defining attributes: supports normative understanding, rights awareness, and practical response capacity. Empirical referents: attitudes toward practice boundaries, personal safety, prescription authority, multi-site practice, and rights protection. |
| 52 | When patients take the initiative to audio-record a clinical consultation | Inge Renske van Bruinessen, 2017; cross-sectional study; Netherlands | Investigated oncologists' and nurses' experiences and views regarding patients' audio-recording of consultations, including medico-legal risk, communication effects, privacy, trust, and legal uncertainty. | Defining attribute: supports law-based situational practice. Empirical referents: responses to patient recording in terms of cooperation, communication, risk perception, and privacy protection. Consequence: potential influence on clinician–patient communication and trust. |
| 53 | Limitations in health professionals' knowledge of end-of-life law: a cross-sectional study | Ben P. White, 2024; cross-sectional study; Australia | Examined end-of-life law knowledge, legal attitudes, legal experience, and continuing education among doctors, nurses, medical students, and allied health professionals. | Conceptual boundary: distinguishes self-rated legal knowledge, actual legal knowledge, legal attitudes, and legal experience. Defining attribute: supports normative understanding. Antecedents: CPD training, practice experience, and confidence in applying law. |
| 54 | The association between clinicians' legal literacy and the service quality of primary healthcare: evidence from the Greater Bay Area study, China | Fang Xie et al., 2025; cross-sectional study; Mainland China | Examined the association between legal literacy and service quality among healthcare workers in primary healthcare institutions. | Defining attributes: provides direct evidence for the structure of legal literacy, identifying knowledge, attitude, and compliance as components. Empirical referents: legal knowledge, legal attitude, and law-abiding behavior. Consequence: higher legal literacy is associated with better primary healthcare service quality. |
| 55 | Legal Awareness and Practices of Female Genital Mutilation/Cutting (FGM/C) among United Arab Emirates Medical Practitioners | Shamsa Al Awar, 2023; cross-sectional study; United Arab Emirates | Investigated UAE healthcare practitioners' awareness and practice related to complications, legal regulations, cultural acceptability, attitudes, and reporting obligations regarding female genital mutilation/cutting. | Defining attributes: supports normative understanding, value internalization, and law-based situational practice. Antecedent: cultural tradition. Empirical referents: awareness and attitudes regarding legal regulations, reporting obligations, complications, and prohibitive norms. Consequence: insufficient legal awareness may lead to tolerance or inappropriate acquiescence in unlawful medical practices. |
| 56 | Perception of nurse prescribing among nurses and psychiatrists in a developing country: A cross-sectional survey | Ashish Badnapurkar, 2018; cross-sectional study; Qatar | Surveyed nurses' and psychiatrists' attitudes toward nurse prescribing, clinical responsibility, legal responsibility, training needs, and supervision models, comparing perceptions of legal and clinical responsibility. | Conceptual boundary: distinguishes legal responsibility, clinical responsibility, and professional autonomy. Defining attributes: supports normative understanding and value internalization. Antecedents: legal training and clarity of role boundaries. Empirical referents: attitudes toward clinical responsibility, legal responsibility, supervision, and training needs. |
